# Peritesticular xanthogranuloma mimicking testicular cancer: case report

**DOI:** 10.1097/MS9.0000000000005806

**Published:** 2026-10-22

**Authors:** Amhasilasie Ewunetu Ageze, Haymanot Alemayehu Bekele, Adugna Atnafu Atalay, Gobezu Assefa Getahun

**Affiliations:** a Department of Surgery, Kobo Hospital, Kobo, Ethiopia; b General Practice, Pugnido Refugee Camp Health Center, Gambella, Ethiopia; c Department of Pathology, Woldia University, Woldia, Ethiopia; d Department of Basic Sciences, New Anglia University, Valley, Anguilla

**Keywords:** testicular cancer, testis, xanthogranulomatous

## Abstract

**Introduction and importance:**

Peritesticular xanthogranulomatous inflammation (XGI) is a rare cause of scrotal swelling that clinically mimics testicular cancer. It is characterized by tissue destruction and replacement by a prominent cellular infiltrate of lipid-laden macrophages. The pathogenesis remains unclear. Surgical management remains the mainstay of treatment.

**Case presentation:**

We report the case of a 32-year-old man who presented with progressively enlarging left testicular swelling for 3 years. Physical examination revealed a large, non-tender left scrotal mass with negative trans-illumination. Ultrasonography revealed a heterogeneously hypoechoic scrotal mass. An orchiectomy was performed to exclude malignancy. Further pathologic examination revealed a dominant infiltrate of foamy macrophages interspersed with a minor component of lymphocytes, plasma cells, and multiple abscess foci in the peritesticular tissue. The patient had a stable postoperative course. He had no complaints during subsequent follow-up visits.

**Clinical discussion:**

Peritesticular XGI is an exceptionally rare condition with an unclear pathogenesis. It can closely mimic testicular malignancy clinically and radiologically, making a preoperative diagnosis difficult. This case highlights its unique peritesticular distribution and underscores the diagnostic challenge it poses, adding valuable insight to the limited literature.

**Conclusion:**

This case highlights the rarity and diagnostic challenges of xanthogranulomatous processes, particularly when differentiating them from malignancy. A meticulous and systematic approach is essential for the evaluation and management of such cases. Further research is needed to provide evidence-based recommendations specific to the diagnosis and treatment of peritesticular xanthogranulomas.

## Introduction

Xanthogranulomatous inflammation (XGI) is an uncommon variant of chronic inflammation and a well-established pathological entity^[^1^]^. It is characterized by xanthogranuloma, which is composed of lipid-laden foamy macrophages, lymphocytes, and plasma cells with variable numbers of foreign-body giant cells that lead to the destruction of the tissue in the affected organ^[^2^]^. XGI is known to involve various organs; however, the most common sites are the gallbladder and kidney, called xanthogranulomatous cholecystitis and xanthogranulomatous pyelonephritis, respectively^[^2–4^]^. Although XGI was first reported in the genitourinary tract^[^5^]^, peritesticular xanthogranuloma remains rarely reported^[^6^]^. Herein, we report a case of biopsy-confirmed peritesticular xanthogranuloma that was surgically managed to exclude malignancy.

This case report has been prepared in line with the SCARE guidelines checklist 2025^[^7^]^.

HIGHLIGHTS

- Rarity and knowledge gap: Peritesticular xanthogranuloma is an exceptionally rare variant of xanthogranulomatous inflammation, characterized by sparing of the testis and epididymis, with very few cases reported.
- Unclear pathogenesis: The exact mechanism remains uncertain, although obstruction, chronic infection, immune dysfunction, and abnormalities in lipid metabolism have been proposed.
- Diagnostic challenge: Preoperative investigations, including imaging, tumor markers, and fine needle aspiration, are of limited value, making differentiation from testicular malignancy difficult and often necessitating an orchiectomy for definitive diagnosis.

## Case presentation

A 32-year-old male patient presented with left scrotal swelling that had gradually increased in size over a period of 3 years, accompanied by dull pain. He denied urethral discharge, urinary symptoms, constitutional symptoms, or any history of trauma. His past medical and surgical history was unremarkable. Physical examination revealed a large, non-tender, left scrotal mass with negative trans-illumination and a firm-to-hard consistency, measuring 12 cm in maximum diameter. No skin involvement or sinus discharge was observed. Inguinal lymph nodes were not enlarged. The right testis had a normal appearance.

Laboratory investigations revealed a leukocyte count of 9 × 10^3^/µL (neutrophils 67%), hemoglobin 14 g/dL, hematocrit 31%, and platelet count 310 000/µL; urinalysis and random blood sugar tests were normal. The serum alpha-fetoprotein level was 8.2 ng/mL, which was within normal limits. Ultrasonography revealed a heterogeneously hypoechoic scrotal mass.

Left inguinal orchiectomy was performed to exclude malignancy. A single inguinoscrotal incision was made to deliver a large mass without violating the overlying skin or tunica vaginalis. The spermatic cord and vascular pedicle were ligated at the level of the internal inguinal ring, and the mass was completely excised. The surgical wound was irrigated with copious saline and closed primarily. The patient had an uneventful postoperative course and was discharged on the third postoperative day with oral antibiotics and instructions for wound care. The resected specimen measured 12 cm × 10 cm × 8 cm and weighed 650 g (Fig. 1)

**Figure 1.**
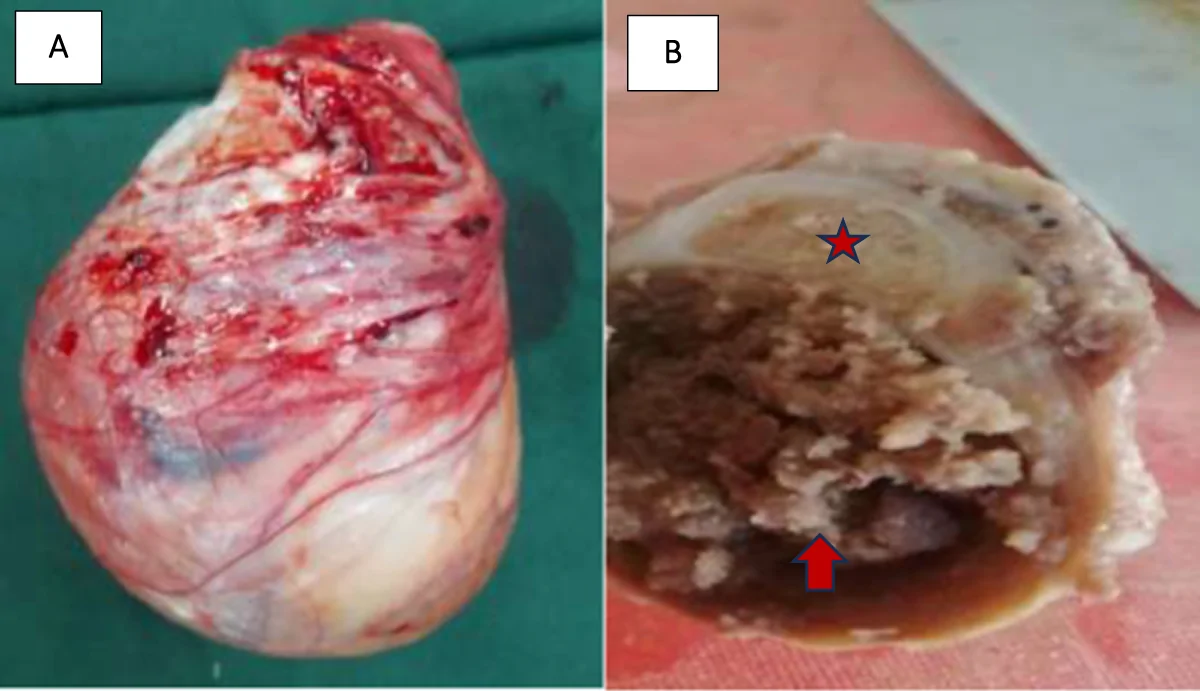
Gross pathologic images. (A) External appearance of the orchiectomy specimen. (B) Cut surface of the orchiectomy specimen demonstrating a cystic cavity consisting of gray-white friable tissue (arrow) and normal testicular parenchyma (star).

On gross examination, the cut surface revealed an ill-defined, dark-yellow, necrotic tissue-filled mass, surrounded by a thick fibrous capsule that compressed the testis toward the periphery, causing slight effacement (Fig. 1). Histologic examination of the peritesticular sections reveals a fibrous cyst wall enclosing amorphous, granular, necrotic debris, numerous cholesterol clefts, and a mixed inflammatory infiltrate composed of foamy macrophages, lymphocytes, and eosinophils (Fig. 2). Notably, the adjacent testicular parenchyma and epididymis show no significant histopathologic abnormalities, with no involvement or destruction of the seminiferous tubules.

**Figure 2.**
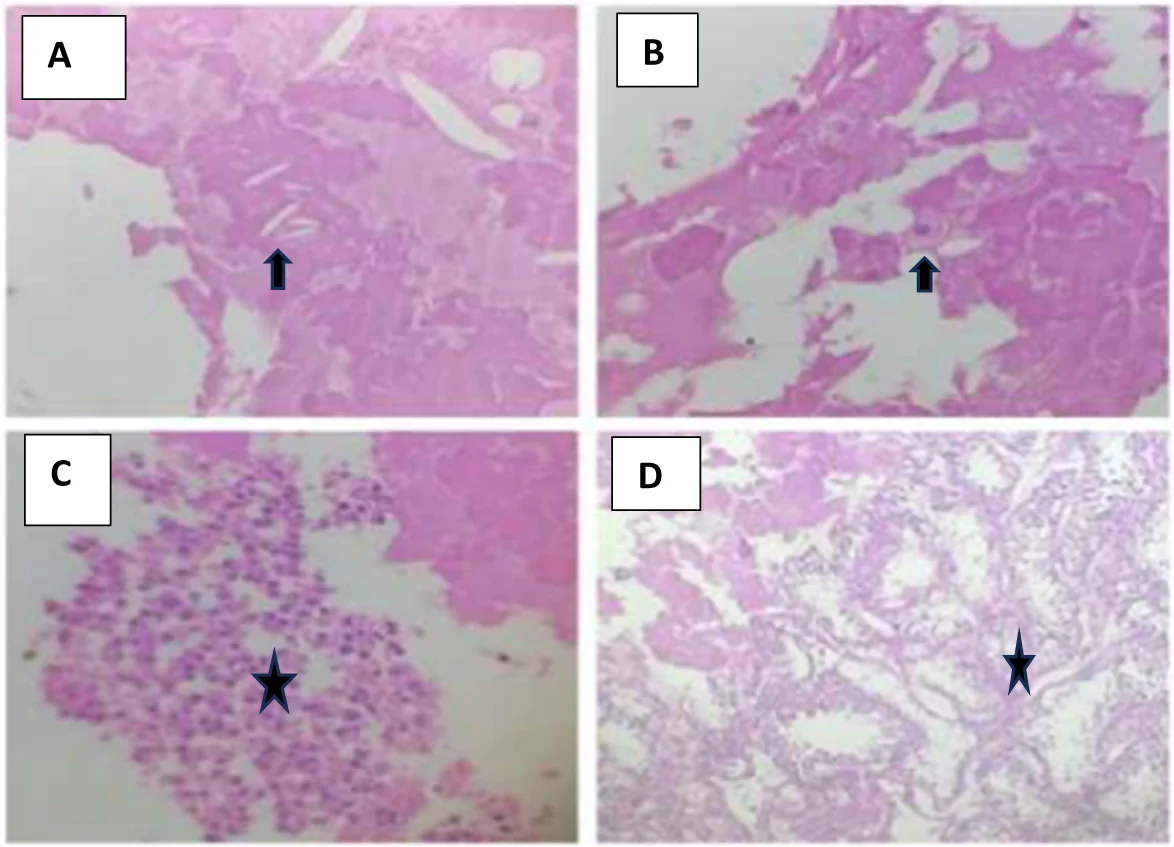
Hematoxylin- and eosin-stained microscopic images. Cholesterol clefts (A), lipid-laden macrophages (B), mononuclear inflammatory cells (C), and normal testicular parenchyma (D) are demonstrated at 10× magnification.

## Discussion

XGI is a benign, destructive condition that can produce tumor-like lesions that mimic malignant processes^[^8,9^]^. XGI is classically described as a variant of chronic inflammation characterized by tissue destruction and replacement, with a prominent cellular infiltrate of lipid-laden macrophages^[^10^]^.

Although the exact pathogenesis of XGI remains unclear, several studies suggest that multiple factors contribute to its development. The primary mechanisms proposed include mechanical obstruction, chronic infection, immune system dysfunction, and abnormalities in lipid metabolism^[^5,8,10,11^]^.

In one study, which included a case series of four patients along with a review of 38 reported cases of xanthogranulomatous epididymo-orchitis, diabetes mellitus was the most common predisposing factor, supporting the possible contribution of immune dysfunction^[^12^]^. In another report, trauma was identified as an inciting event, further highlighting the multifactorial nature of triggers associated with XGI^[^13^]^. However, in our case, the patient had a normal random blood glucose level, no history of immunocompromising medical conditions, and denied any prior trauma, making the underlying trigger for XGI unclear.

Mechanical obstruction has been proposed as one of the major contributing host factors in the development of XGI. This mechanism is considered particularly plausible in organs with ductal or tubular structures, such as the kidney, biliary tract, pancreas, epididymis, and fallopian tubes^[^2,8,12^]^. For instance, in cases of xanthogranulomatous orchitis (XGO), mechanical obstruction of the urinary tract is thought to contribute to urinary tract infection, followed by retrograde reflux of urine into the male genital tract, ultimately initiating the inflammatory process^[^12^]^. In our case, however, the inflammatory process was confined to the peritesticular tissue, sparing both the epididymis and testis, suggesting the possibility of an alternative underlying mechanism.

The characteristic pathologic features of XGO include infiltration of the interstitial tissue, with compression of seminiferous or epididymal tubules, destruction of the testicular parenchyma, and its replacement by a diffuse proliferation of foamy macrophages. The inflammatory infiltrate is typically composed of lymphocytes, plasma cells, neutrophils, and eosinophils^[^10,12^]^. Similarly, in our case, the pathological findings revealed a predominant infiltrate of foamy macrophages, with a minor component of interspersed lymphocytes and plasma cells. However, the inflammatory process was confined to the peritesticular tissue, with no involvement of the testis or epididymis, and no evidence of seminiferous tubule destruction. This distribution is particularly intriguing, as it differs from the typical histological pattern seen in XGO, which usually involves the testicular parenchyma. Notably, our patient did present with testicular swelling, clinically mimicking XGO, yet the histological findings confirmed a distinct peritesticular xanthogranuloma, a rarely reported condition^[^6,13^]^.

This structural distribution directly speaks to a broader academic debate regarding whether xanthogranulomatous lesions involving the scrotal sac represent a primary paratesticular process or are simply secondary extensions of severe inflammation originating inside the testicular or epididymal compartments. Our case provides evidence of the existence of a distinct subset of XGI limited to the peritesticular region. The definitive sparing of both the testis and epididymis in our patient directly contradicts the prevailing pathophysiology detailed in most documented case series, where retrograde ductal breakdown serves as the baseline inflammatory catalyst^[^12^]^. This complete absence of intragonadal or epididymal tissue involvement underscores the clinical importance of recognizing primary paratesticular pseudotumors to successfully implement testis-sparing, organ-preserving surgeries and prevent unwarranted radical inguinal orchiectomies^[^17^]^.

Preoperative diagnosis of XGO is challenging because its clinical and radiologic features can closely mimic testicular neoplasms^[^9,14^]^. Although scrotal ultrasonography may help exclude other causes of acute scrotal pathology, no specific sonographic features reliably distinguish XGO from malignancy. Similarly, magnetic resonance imaging and radionuclide testicular scanning have limited discriminatory value and may not reliably differentiate XGO from testicular neoplasms^[^9,14,15^]^. In our patient, the presence of a firm-to-hard scrotal mass and a heterogeneous hypoechoic lesion on ultrasonography likewise raised concern for malignancy. Preoperative investigations, including fine-needle aspiration and serum tumor markers, are also of limited diagnostic value. Reliable diagnosis of XGO by fine-needle aspiration has not been established, while serum tumor markers may remain normal, as in our patient^[^9,14,16^]^. Thus, definitive diagnosis generally relies on histopathologic examination.

The diagnostic uncertainty has important implications for surgical management. Because malignancy often cannot be excluded preoperatively, radical inguinal orchidectomy has been the predominant treatment reported in the literature, with only rare cases managed by testis-sparing enucleation^[^6,13^]^. Intraoperative frozen-section examination may provide an opportunity to assess the lesion and guide the extent of resection when the testicular parenchyma appears uninvolved^[^12^]^. In our case, orchidectomy was performed because malignancy could not be confidently excluded before or during surgery. Given the complete preservation of the testis and epididymis on histopathologic examination, recognition of primary peritesticular XGI in future cases may support consideration of organ-preserving surgery in appropriately selected patients, particularly when intraoperative pathology can establish the benign nature of the lesion.

## Conclusion

This case highlights the rarity and diagnostic challenges of xanthogranulomatous processes, particularly in differentiating them from malignancy. A meticulous and systematic approach is essential in the evaluation and management of such cases. Further research is needed to develop evidence-based recommendations specific to the diagnosis and treatment of peritesticular xanthogranuloma.

## Data Availability

The data supporting the findings of this case are available upon request to the corresponding author.
